# DIPLOMAT: multi-animal tracking with efficient manual editing

**DOI:** 10.1101/2025.08.11.669786

**Published:** 2025-08-26

**Authors:** Isaac Robinson, George Glidden-Handgis, Neekesh Panchal, Nathan Insel, Travis J. Wheeler

**Affiliations:** 1 University of Arizona; 2 Wilfrid Laurier University

## Abstract

Recent advances in computer vision have enabled the development of automated animal behavior observation tools. Several software packages currently exist for concurrently tracking pose in multiple animals; however, existing tools still face challenges in maintaining animal identities across frames and can demand extensive human oversight and editing. Here we report on DIPLOMAT, a Deep learning-based, Identity-Preserving, Labeled-Object Multi-Animal Tracker, which implements automated algorithms to improve identity continuity, supplemented by an efficient human interface to help eliminate remaining errors. DIPLOMAT is designed to perform multi-animal tracking by building on the per-frame pose prediction models of two state-of-the-art tools, DeepLabCut and SLEAP, applying novel methods to tolerate occlusion and preserve animal identity across frames. Notable features include leveraging model-derived positional probabilities to compute independent maximum probability traces across frames of a video, use of video-specific skeletal constraints, and implementation of an efficient user interface for resolving errors. On the MABe mouse tracking benchmark, automated tracking with DIPLOMAT reduces body identity swaps by *>*75%, while remaining errors are easily eradicated with manual correction.

## Introduction

During social behavior, one individual’s movements can depend on subtle, moment-to-moment changes in another’s facial expressions or posture. Our ability to fully understand social interaction – from biological mechanism to its role in health or ecology – may depend on methods to monitor the complexity of these movements across multiple animals. Computer vision advances have made this increasingly possible, but there are still many practical limitations. A particular challenge has been in keeping track of identities: where standard algorithms excel at identifying the locations of body parts in a given image, they often still falter in maintaining a consistent identity for the owner of those parts across a recorded video.

Several tools for multi-animal tracking have recently become available, including ID tracker (Romero-Ferrero et al., 2019), AlphaTracker (Chen et al., 2020), TRex (Walter and Couzin, 2021), SLEAP (Pereira et al., 2022), multi-animal DeepLabCut (DLC; Lauer et al. 2022), and PrecisionTrack (Coulombe et al., 2024). Three of these, DLC, SLEAP, and AlphaTracker have caught-on as particularly popular and powerful tools for tracking dyads and triads of rodents using a single-camera video source (Shemesh and Chen, 2023; Luxem et al., 2023; Bordes et al., 2023; Padilla-Coreano et al., 2022). Each of these three tools proceed by first assigning identities on individual frames, then stitching them together across frames using various similarity-matching methods. For assignments within each frame, DLC follows a “bottom-up” (parts-to-bodies) approach, predicting probability distributions for body parts then assembling them into per-animal skeletons via graph/geometry constraints. AlphaTracker follows a “top-down” (bodies-to-parts) approach, first detecting animal bounding boxes, then running a single-animal pose estimator within each region. SLEAP supports both bottom-up and top-down part assignments. To stitch identities across frames, the tools use different but related methods: DLC uses spatial proximity and pose similarity, AlphaTracker uses overlap of bounding box (body) and probability distribution (body parts), and SLEAP likewise uses pose-space similarity along with motion models (optical-flow/Kalman style predictions) to account for frame-to-frame displacement. PrecisionTrack uses a Dynamic Kalman Filter as the motion model. In cases where tracked bodies are visually distinct, additional algorithms can be applied; e.g., in DLC users can specify identifying features, or use a transformer-based “reID” method that automatically detects visual distinctions. Each of these approaches to frame-by-frame continuity seems theoretically viable, yet empirically they are still known produce identity persistence errors, in which either individual body parts or entire skeletons are swapped between individuals. Errors are particularly common when animals are near or on top of one-another, causing full body swaps, or skeletons spanning both bodies (“chimeras”). While post-tracking user-edits can help correct for these, editing can be laborious.

Here, we introduce DIPLOMAT (Deep learning-based, Identity-Preserving, Labeled-Object Multi-Animal Tracker), which seeks to improve identity accuracy and persistence in multi-animal tracking. Rather than introducing an entirely new tool, we designed DIPLOMAT as a method capable of leveraging and expanding on the effective per-frame pose prediction results of other tools. DIPLOMAT currently uses models trained by either DLC or SLEAP as the basis of per-frame pose prediction, then applies a distinct set of downstream algorithms to make predictions about body part location and animal identity. Importantly: the goal of DIPLOMAT is not to address all possible multi-object tracking options, but to specifically introduce algorithms and editing tools that improve identity preservation independent of visual distinctions between objects (i.e., can be applied to tracking nearly identical animals). We also treat characterization of social interaction based on multi-animal pose estimation as outside of the scope of the software; other tools that address automated social classification, primarily in mice, include JAABA (Kabra et al., 2013), MARS (Segalin et al., 2021), MoSeq (Weinreb et al., 2023), and SimBA (Goodwin et al., 2024),.

DIPLOMAT is developed with two components in mind. The first (Track) implements algorithms that help automatically track body parts of multiple animals while minimizing identity errors. The second (Interact) employs an intuitive and efficient user interface for rapid editing of multiple body parts across video frames, with the ability to re-integrate these edits to a quickly and smoothly re-track.

DIPLOMAT casts the tracking problem as one in which bodies and their parts have been produced by a Markov process, and can thus be addressed with a hidden Markov model (HMM). DIPLOMAT treats the per-frame pose labeling confidence values from (DLC or SLEAP) neural-network models as the source of the Markov model’s positional observation probabilities. It then applies the HMM Viterbi algorithm (Forney, 1973) to recover a maximum probability trace across the full history of frames in a video. The DIPLOMAT Markov model includes transition probabilities that incorporate both body motion and body part (skeletal) connections, and enacts a novel frame-by-frame mechanism for splitting probability fields to support mutually independent traces. The full-trace perspective of Viterbi allows DIPLOMAT to avoid making identity switch errors that may occur under greedy (locally-aware) methods of stitching identity between neighboring frames.

DIPLOMAT methods are guided by 4 assumptions: **(1) Bodies don’t teleport**: neighboring video frames (past and future) can be used to inform probability distributions for current body part locations. **(2) Body parts stick together**: skeletal information can further inform a posterior probability of body part location. **(3) Bodies will be easier to distinguish in some frames**: some frames can serve as an anchor, or “ground truth”, and those anchored assignments can influence the labeling of neighboring frames. Finally, **(4) in a given recording, there is typically a pre-set number of bodies**: probability distributions can be sliced into a fixed number of independent probability clusters. Although not all of these assumptions will be true for every application, they cover a wide scope of animal behavior and neuroscience protocols.

In this paper we provide a detailed description of the algorithms supporting DIPLOMAT tracking, and demonstrate that DIPLOMAT’s automated methods reduce identity-swaps in a standard benchmarks dataset (MABe; (Sun et al., 2022)). We selected DLC and SLEAP in part due to their popularity, and also because their bottom-up approaches allow DIPLOMAT to track using the same models. We further describe a novel *Interact* interface that allows users to dynamically and efficiently correct inferred body part locations in frames. A key feature of Interact over other manual editing tools is that it uses probability maps from automated processes to facilitate single- or multi-body part editing. Whereas other tools discard per-frame part placement probability distributions during the course of tracking, DIPLOMAT explicitly retains them for use in semi-automated support of manual correction. Interact includes the ability to dynamically re-trace (re-run the independent-trace Viterbi algorithm) across frames, thereby incorporating user-made edits.

## Results

### Tracking process

DIPLOMAT can be installed using the DIPLOMAT PyPi package (https://pypi.org/project/diplomat-track/). To use DIPLOMAT for tracking, a user must first train a pose inference model. Currently, DIPLOMAT supports models trained using the open-source packages, DLC and SLEAP (bottom-up). Installation and training method for those tools have been detailed elsewhere (Pereira et al., 2022; Lauer et al., 2022).

**Figure 1.**
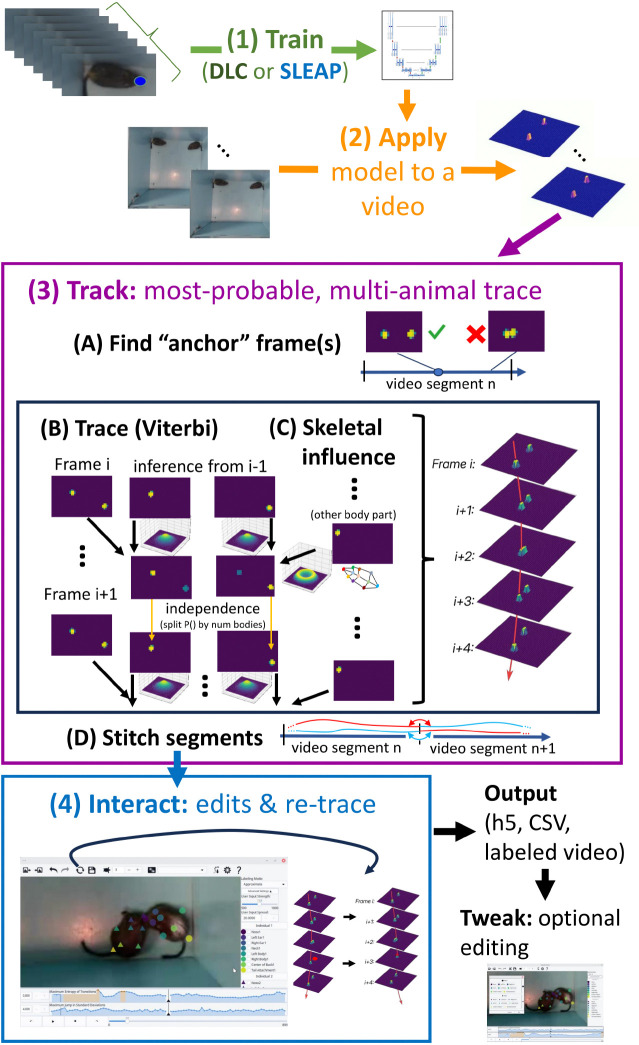
Flowchart illustrating the sequence of steps involved in multi-animal tracking using DIPLOMAT. Step 1 (top): Either SLEAP or DLC is used to train a model by manually labeling frames. Step 2: DIPLOMAT applies SLEAP or DLC inference functions to generate frame-by-frame probabilities on a video. Step 3: DIPLOMAT segments the video by identifying “anchor-frame” locations where animals are spatially separated (A), then combines a Viterbi trace (B) with skeletal constraints (C) to generate a map of transition probabilities. For each frame, independence between tracks for different individuals is imposed by splitting transition probabilities based on path-probability dominance. Segments are then stitched together (D), using a Hungarian algorithm mapping of probability map peaks to preserve identity between segments. Step 4: Errors in tracking can be identified and corrected using the Interact interface, which leverages the same probability maps to assist with single- or multi-body part edits, and allows the automated algorithms to be re-run. The output of Interact is a table of x-y position data across all body parts and individuals. These can be further edited using a paired-down interface Tweak. See Methods for details of all steps.

Given a video and a trained model, DIPLOMAT’s Track module performs per-frame pose inference using a custom implementation of the inference engine appropriate for the trained model. Following per-frame pose inference, DIPLOMAT seeks a trace for each body part (keypoint) that maximizes trace probability under a model that mixes pose inference probability fields with movement distance probabilities supported by skeletal influence. Following automated tracing, the user may *Interact* with the resulting trace to correct errors. See Figure 1 for an overview, and Methods for details.

### Validation

Quality metrics were computed for DIPLOMAT in conjunction with the standalone SLEAP and DLC. As a benchmark, we used the MABe

“Mouse triplet” data set as a source of ground-truth standardized videos (Sun et al., 2022). A prelabeled training set of fifteen 60-second videos from MABe was used to train models (27,000 frames). Tracking was performed, using each tracking tool, across 60-second evaluation mouse triplet videos provided by MABe, filtering to retain only the 54 videos in the dataset that contained continuous, ground-truth position data for each body part of interest (i.e., each frame contained each body part). Tools were tested under two body architectures: one included 10 body parts (nose, ears, neck, forepaws, hind paws, center-back, and tail base) and the other only 5 body parts (nose, ears, center-back, tail-base). We refer to these as BPM-10 and BPM-5 respectively. Metrics were computed using the MotMetrics analysis package (Heindl, 2017); in particular, body swaps are computed using the *num_switches* metric. The set of videos proved to be an adequate (if relatively minimal) sample to detect differences between software tools, as over 40% of videos (24 and 23 for BPM-10 and BPM-5 respectively) induced at least one tracker to produce at least one one “full body swap” (i.e., where most body parts of an animal moved to another body), while over 70% (39 and 40 in for BPM-10 and BPM-5 respectively) induced at least one body part swap in at least one tracker.

**Figure 2.**
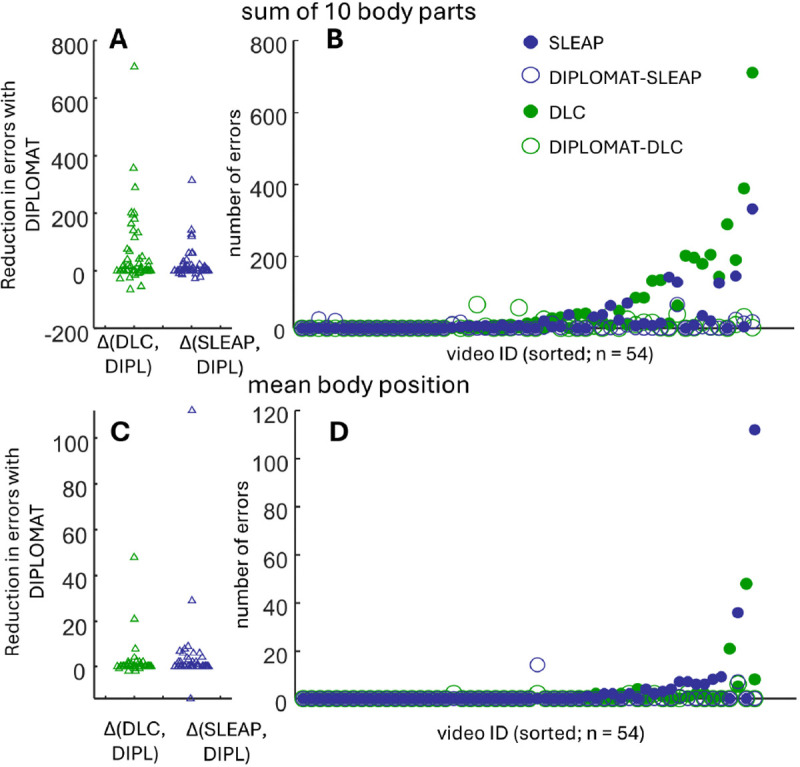
Comparison of body-swap events using the automated-only components of DIPLOMAT (Track) against automated tracking using stand-alone SLEAP or DLC. (A) Swarm plot showing the improvement, when using DIPLOMAT, in the number of cases in which any of 9 body parts across any of 3 animals switches to an incorrect individual; each point is computed for a single video by subtracting body swap errors using DIPLOMAT-DLC from DLC alone (green), and DIPLOMAT-SLEAP from SLEAP alone (blue). (B) Data across all 54 MABe triplet videos, sorted according to maximum number of swaps in either DLC or SLEAP (solid green and blue circles respectively). Although use of DIPLOMAT (open circles) occasionally resulted in more body swaps (appearing above the slope), in the majority of cases DIPLOMAT reduced or eliminated body swaps (open circles below the slope of filled circles) (C&D) Same as A&B but measuring whole-body swaps (calculated as mean body position).

DIPLOMAT improved tracking relative to both DLC and SLEAP when using either BPM-10 or BPM-5. Using the BPM-10, the incidence of body-swaps across individual body parts was lower when DIPLOMAT was used against DLC or SLEAP (Figure 2 A&B; top row of Tables 1; Wilcoxon signed rank test for zero median). Strong reductions were also observed in full body swaps (Figure 2C&D; top row of Table 2). Using BPM-5, body part identity swaps were reduced compared to standalone DLC (Table 1), and full body swaps reduced relative to SLEAP, with a strong statistical trend against DLC (Table 1). DIPLOMAT also improved scores across other important measures. ID recall – the proportion of body parts (Table 1) and bodies (Table 2) that DIPLOMAT correctly identified – was consistently higher with DIPLOMAT. DIPLOMAT also improved whole-body ID precision, a measure inversely related to the number of false-positives, and for individual body parts showed improvements over SLEAP but not DLC. Finally, DIPLOMAT tended to show higher proximity of points identified relative to ground-truth points (MOTP) when compared against the same model used in stand-alone SLEAP, with only minor differences observed compared to the same standalone DLC model.

#### Potential for errors

There are several ways in which Track may remain insufficient for body part location inferences. One way that differs from status-quo methods is if a body leaves and then quickly returns to a location, independent-trace Viterbi may maintain the body part locations between the movement frames, treating it as a more probable option than following the fast moving animal across frames. In our experience, this happens extremely rarely. Another is common to all tools: when animals overlap with one-another (consequently pushing entropy of the probability distributions low) there is an increasing chance that probability fields will be split in the wrong direction, causing an identity swap. Although these challenges can be addressed with further algorithmic changes, without use of multiple cameras, it is difficult to envision any tool that perfectly tracks multiple animals without cases of identity confusion. We therefore develop a follow-up tool Interact that provides a memory efficient and intuitive user interface to review, edit, and re-track.

### Interact: editing tracks using the DIPLOMAT interface

**Figure 3.**
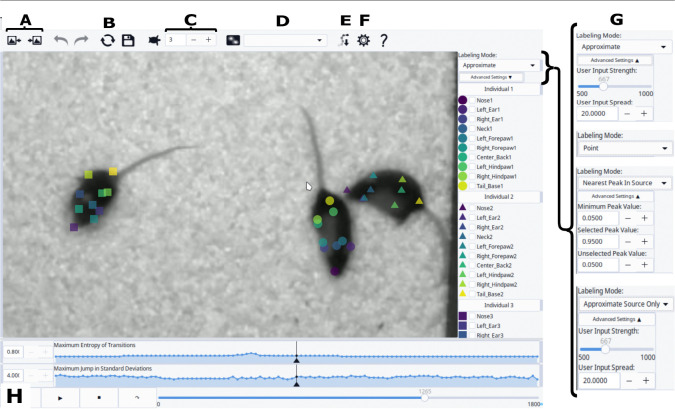
Interact can be used to edit body parts after automated tracking. Image includes letter labels associated with interface tools, as follows: (A) “Frame skip” advances to the next (or prior) section of video where tracking errors are more likely, as measured by entropy and frame-to-frame peak jumps (see H). (B) “Re-run” quickly re-applies automated tracking algorithms, incorporating the edits. (C) “Hover-track speed”: adjusts video playback speed when holding down the CTRL key to continuously label (i.e., using “hovering-track” functionality). (D) The user can optionally overlay the probability fields produces by the inference engine, perhaps to inspect the cause of labeling errors. The drop-down box allows the user to select which body part (e.g. all noses) will be shown. (E) “Export” creates a CSV file with all inferred body part positions throughout the video. (F) “Settings” allows user to adjust marker sizes and colors. (G) “Labeling mode” switches between label modes, which are optimized for different conditions (e.g., editing single versus multiple body parts, adjusting for poor probability maps, correcting identity swaps versus occlusion, etc.). Each labeling mode has adjustable parameters. (H) Plots showing frame-by-frame measures of tracking confidence, based on transition matrix entropy (related to animal proximity) and distance that peaks moved. User-defined thresholds help to flexibly identify concerning sections of the video.)

We developed DIPLOMAT’s Interact to allow users to quickly correct errors remaining after automated analysis in the Track stage. The Interact workflow can be summarized as following three steps, often repeated across multiple cycles when reviewing a video: (1) review tracked positions, (2) manually edit body part positions, and (3) re-run automated trace routines. Figure 3 provides a labeled screenshot of the Interact interface.

To support rapid identification of potential errors, DIPLOMAT computes two per-frame measures that may highlight frames with a high risk of error: (i) maximum transition entropy and (ii) maximum jump. Maximum entropy is a measure of the extent to which a body part instance in one frame may have more than one viable candidate for pairing in the next frame; a low value indicates that individuals are well separated, while a high value indicates that at least two animals are in close proximity. The jump distance is calculated by determining the Euclidean distance of each body part instance in the frame to its location in the next frame, then dividing that by the standard deviation of movement for that body part; the largest of these values is the *Maximum Jump in Standard Deviations*.

When using the Interact interface, the tracked video can be played with labels overlaid; the user can watch the entire labeled video, or may choose to skip to the next/previous point in the video with above-threshold entropy/jump values. After identifying a tracking error, the user can correct the error with either precise or broad actions. One option is to select a body part, then click to place it on the screen, optionally placing the part continuously across many frames while pressing the CTRL key. Another option is to place/correct many body parts concurrently – this is performed by selecting a set of parts (or an entire body), then clicking onto the screen. In both cases, DIPLOMAT provides a labeling mode (upper right corner of window, Figure 3), that often substantially improves speed of placement. In the “Nearest Peak in Source” labeling mode, each body part label finds the matching probability peak nearest to the user mouse placement, and assigns the part to that location. This can be particularly useful in cases where the initial automated tracking has fully swapped two bodies (i.e., the user is pulling points away from one set of local minima to another). Other labeling modes, such as “exact” and “approximate”, are better suited for single-body part labeling, such as following a misplaced body part.

After one or more frames has been edited, the user can re-run the automated tracing algorithm. Prior to re-running the Viterbi maximum trace probability algorithm, probability maps are updated to treat edited locations as 100% confident, so that the trace is forced to use that placement. As a result, it is often only necessary to edit one or a few frames to fix a body swap.

## Discussion

The DIPLOMAT software package offers a new set of algorithms and interface tools for tracking multiple, freely moving animals in single-camera, controlled environments. We show that these methods reduce body part and full-body swaps compared with other state-of-the-art packages, DLC and SLEAP. DIPLOMAT also produced other improvements, particularly in identity recall and, when compared against SLEAP, both precision and MOTP (i.e., tracking distance from ground truth). We further described a novel, human interface tool, Interact, that improves manual editing of automatically-computed traces. Interact includes the ability to dynamically re-trace (re-run the independent-trace Viterbi algorithm) across frames, thereby incorporating user-made edits.

Beyond reducing body swaps, DIPLOMAT can improve multi-animal tracking by ensuring that all body parts are accounted for. When using stand-alone convolutional neural network tools, cases of occlusion or unique body angles often lead to lost or missing body parts. The DIPLOMAT algorithms ensure object permanence, with the combination of Viterbi and skeletal probabilities smoothly filling gaps when the body part is not clearly visible. If desired, users can still omit these tracking points by removing “occluded state” points. While there can be risks in inferring keypoint locations that can’t be “seen” in a given video frame, the logic is consistent with unconscious inferences (to use the terminology of Helmholtz) used by all known evolved visual systems; i.e., visual knowledge is built from wedding sensory data to an internal model. Risks are also mitigated by preserving frame-by-frame probability estimates from Track. This particular “internal model” approach is different and in some ways less powerful than others that have been used; e.g., inferences based on trained mappings to 3D pose data (Dunn et al., 2021). However, they are also compatible, and could potentially be used in parallel. Furthermore, trained models depend on the training set, and may have limited external validity across animal strains and species, while algorithms like Viterbi and skeletal information are based on parameters that can be adjusted according to context.

Although our current implementation of DIPLOMAT builds on either SLEAP or DLC, the package is designed to be adaptable to any neural network based tracking tool. Any combination of algorithms that are implemented here – the independent-trace Viterbi, skeletal probability distributions, or manual edit tools – could be added as a layer on top of other tracking methods, or wrapped within higher-level, open-source and proprietary interfaces (e.g., popular packages such as Ethovision or ANY-Maze, Noldus et al. (2001); Spink et al. (2001); Lim et al. (2023)). It is possible, therefore, that individual tools packaged within DIPLOMAT will have value or reach beyond the software as a whole (that the whole is, for many applications, less than the sum of its parts). As an example: the Tweak interface is designed to be highly versatile, and can be easily repurposed for other tracking methods and integrated with other data streams. Tweak offers a simplified method to evaluate and edit tracks from exported CSV files, including swapping body parts or whole bodies between individuals. It is already designed to recognize and convert CSV files generated from DLC or SLEAP, but CSV conversion from other formats would be simple to implement.

Like any open-source software, the current version of DIPLOMAT can be seen as a functional snapshot, and with time and interest can be expected to evolve according to user needs. We have begun to consider several examples: First, the methods currently have no notion of size variability, which could be introduced both by modified neural network approaches as well as dynamic (potentially Markov Model informed) skeletal distances. Second, the Gaussian, spatial transition probabilities are currently static, but minor adjustments to the code could be made to fit an animal’s instantaneous velocity. Third, the code could be adjusted to work with multi-camera methods for improved, 3D posture inferences. While many research questions can be adequately addressed by 2D models, or 3D inferences using single cameras (Karashchuk et al., 2021), there has been increasing interest in creating detailed, 3D pose estimates from multi-camera methods, including in mice (Klibaite et al., 2025), primates (Hayden et al., 2022) and human subjects (Wang et al., 2021). An intriguing challenge in this domain will be adapting Interact for fast, 3D-view human editing.

Together, then, DIPLOMAT both offers an important step toward more accurate and efficient, multi-animal tracking in laboratory conditions, while also providing a set of tools and map for their usage that can be repurposed across many tracking applications.

## Methods

DIPLOMAT achieves multi-animal tracking with low body-swap frequency by casting multi-animal tracking as a problem of finding multiple independent high-probability traces in a system of observations treated as having been produced by a hidden Markov model. The process consists of multiple stages, described in detail below. In brief, they are:

- The user selects a pose inference engine (currently either SLEAP or DeepLabCut), then trains a model following the instructions appropriate for the selected engine. This can be performed in an engine-specific compute environment, with no connection to DIPLOMAT.
- Given a video and a trained model, DIPLOMAT performs pose inference; this yields pseudo-probabilities that each body part is present at each coordinate (pixel) in each frame.
- DIPLOMAT processes the per-frame pseudo-probabilities. It clusters neighboring pixels, computes estimates of body/skeleton size(s), identifies reliably-separable frames (“anchor frames”), and splits video into segments separated by those frames.
- For each segment, DIPLOMAT computes a maximum probability trace starting from the segment’s anchor frame. A trace depends primarily on body part position estimates from the pose engine and the movement (transition) probabilities of the DIPLOMAT Markov model. DIPLOMAT extends this simple one-body-part-trace protocol in two ways: (a) the model is modified such that each body part for an individual is influenced by each other body part through structured skeletal constraints and (b) the paths of all individuals are computed and adjudicated concurrently, to ensure that paths remain independent (see below for both adjustments).
- The result is a set of mutually independent traces within each segment, one for each individual tracked animal. Each segment can be computed independently, enabling efficient parallelism. DIPLOMAT stitches segments together so that identity is preserved across segments.
- A user may Interact with the tracking results using an intuitive graphical user interface that is designed to enable rapid identification and correction of tracking errors.
- An additional, optional tool, Tweak, provides an editing interface that can used on the final CSV table outputs for point-and-click edits or ID swaps.

### Pose inference engines

DIPLOMAT implements pose inference for models trained using either of two popular tools, SLEAP (Pereira et al., 2022) and DeepLabCut (DLC; (Lauer et al., 2022)). DIPLOMAT is run on the command line by providing a trained model and a video. Because DIPLOMAT contains an independent implementation of the SLEAP/DLC inference architectures, inference with DIPLOMAT can be performed in a computer/environment that is distinct from the SLEAP/DLC training environment. DIPLOMAT’s inference engine uses the Open Neural Network Exchange (ONNX) format and is executed via ONNX Runtime.

### Initial processing

#### Sparse positional probabilities

DLC and SLEAP neural network models compute values N(i,b,x,y) corresponding to the observation of each body part type b at each pixel (x,y) of each frame i. The resulting element-wise sigmoid activations can be interpreted as per-pixel pseudo-probabilities 0.0≤P(i,b,x,y)≤1.0.

SLEAP and DLC identify peaks of P(i,b,x,y) values and assign those locations to individuals. DIPLOMAT instead uses the complete matrices to find a good trace across frames. To reduce downstream computational burden, DIPLOMAT immediately prunes low-probability pixels from consideration, retaining only the sparse set of pixels with P(i,b,x,y)≥1e-6. In each frame, sparse pixels are assigned to n clusters using complete linkage clustering by default (Sorensen, 1948).

#### Estimating body sizes

**Figure 4.**
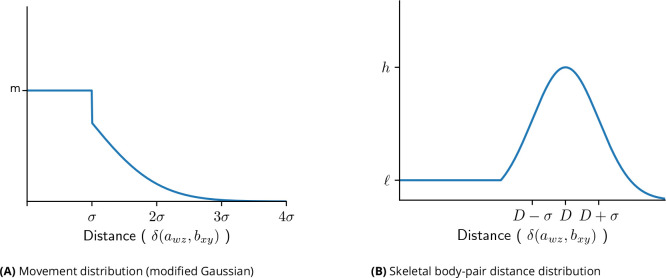
PDFs for (A) body part movement and (B) skeletal influence between body parts, as employed during calculation of a maximum-probability Viterbi trace. (A) The movement PDF is essentially a Gaussian, so that large movements are improbable; because small movements should not be punished relative to total lack of motion, all distances within one standard deviation of movement distance are shifted to be equally probable. (B) The skeletal PDF for a pair of body parts is designed to place high probability that one body part is the appropriate (skeletally-constrained) distance from the other; because bodies are flexible, the PDF maintains some non-zero probability even for distances much smaller than is typical.

DIPLOMAT tracking is based on the assumptions that (i) large changes in position between frames are rare and (ii) all parts of a body tend to remain close to each other, at relatively constant pairwise distances. DIPLOMAT enforces these assumptions without prior knowledge of body sizes or pixel resolution by automatically estimating the size of a body and its skeletal connections. It computes the typical distance between each body-part pair as follows:

- Consider an instance of type a placed at pixel (w,z), represented as $a_{wz}$, and an instance of b placed at pixel (x,y), represented as $b_{xy}$. The distance between these points is the Euclidean distance: $\delta \left(a_{wz},b_{xy}\right)=\sqrt{{\left(x-w\right)}^{2}+{\left(y-z\right)}^{2}}$.
- Each body part type b is represented by n clusters representing the n individuals in the video. For each body part cluster, the location of that instance of body part b is assigned to be the weighted centroid of the cluster (note: this positional estimate is only used in the size estimate stage, not during tracing).
- For a pair of body part types (a,b), we do not know in advance which cluster of a is correctly paired with which cluster of b. This makes it challenging to estimate a typical distance between a and b. DIPLOMAT performs this task by considering each instance of a in frame i, selecting the closest instance of b in that frame, and appending it to a vector of distances for (a,b); this vector eventually stores (a,b) distances across all frames. DIPLOMAT then computes the median and (coefficient corrected) median absolute distance, and uses this as the estimated mean, D(a,b), and standard deviation, σ(a,b). Empirically, the closest point is usually the correct pair, and any errors tend to slightly underestimate the skeletal distance, which is preferable for tracing.
- During tracing, DIPLOMAT uses the expected pairwise distance D(a,b) when assigning a score for placement of body parts a and b in a frame: the score is highest when the distance $\delta \left(a_{wz},b_{xy}\right)$ between the placements is equal to D(a,b), decays to zero as the distance grows much larger than D(a,b) and decays to some constant value >0 for distances smaller than D(a,b). Specifically:
  - Let h be the maximum value assigned when $\delta \left(a_{wz},b_{xy}\right)=D\left(a,b\right)$
  - Let ℓ be the minimum value assigned when $\delta \left(a_{wz},b_{xy}\right)=0$
  - Let $\phi =\frac{{\left(\delta \left(a_{wz},\;b_{xy}\right)\;-\;D(a,\;b)\right)}^{2}}{2\sigma {\left(a,\;b\right)}^{2}}$
  Then the skeletal value assigned to placement of parts $a_{wz}$ and $b_{xy}$ is:
(1) $$K\left(a_{wz},b_{xy}\right)=\left\{\begin{array}{ll} max\left(\ell ,h\cdot e^{-\phi }\right) & \;\text{if}\;\delta \left(a_{wz},b_{xy}\right)<D\left(a_{wz},b_{xy}\right)\\ h\cdot e^{-\phi } & \;\text{otherwise}\; \end{array}\right.$$
  This produces a distribution of scores resembling that shown in Figure 4B, in which a pair of body parts is most likely to have a score h for a distance that matches the pre-computed mode distance, decays to some constant score ℓ for distances as small as zero (corresponding to positioning body parts closer than is common, which can for example happen when an individual curls its body), and decays to zero for distances greater than D(a,b) (corresponding to stretching the body parts farther than they are typically observed).

Note: automatically selected skeletal lengths can be overridden by configuration settings described in the documentation.

#### Finding reliable frames and segmenting video for parallel processing

In order to improve speed, DIPLOMAT splits the video into a series of segments such that (i) a trace can be computed for each segment independently (in parallel), (ii) each segment is at least as long as a minimum segment length threshold σ, and (iii) the split point between two segments occurs at a frame in which individuals are well separated, so that they can be stitched back together. To achieve this, DIPLOMAT computes the reliability R(f) of every frame f; R(f) is a measure of the extent to which individuals are separated in the frame and subtracts the average error across skeletal connection distances. To compute separability of individuals in a frame, DIPLOMAT seeks to assign each body part to an individual in a way that maximizes the coherence of the body parts of the resulting n individuals. One body part group (e.g. all noses) is selected and each instance (cluster) is assigned an arbitrary identity in the range 1 to n. The body part group chosen as seed is the one with the largest number of skeletal connections to other body parts. When multiple parts share the same skeletal degree, the part chosen is the one that has largest minimum distance between pairs of clusters. After this, DIPLOMAT repeatedly selects the next body part b that minimizes the average difference between the distance of b and an already included part a compared with the expected distance D(a,b). Instances of b are assigned to existing individuals using the Hungarian algorithm (Kuhn, 1955) with skeletal body distance scores K(a,b) (equation 1). Once individuals have been constructed, the error across skeletal connection distances is the sum of K(a,b) values across all skeletal connections, divided by n.

After computing R(f) for all frames, DIPLOMAT sorts frames in descending order of reliability, adds the first frame on the sorted list to a fixed_frames list, then scans down the reliability-sorted list until reaching the next frame that is not within σ of an existing fixed_frame, and adds the resulting frame to the fixed_frame list. This list traversal continues until a minimum reliability threshold is reached. At this point, the list of fixed_frames acts as the list of break points between segments. In each segment, the frame g with highest reliability R(g) is the “anchor frame”. The purpose of selecting an anchor frame with high separability between individuals is that each individual in the frame is expected to be correctly resolved – all body parts will be correctly assigned to the same individual. This frame can serve as the starting point for a (Viterbi) maximum probability trace described below.

Within each segment, with n individuals, the anchor frame will contain n bodies (a set of body parts) assigned an arbitrary identifier between 1 and n. There is no reason to expect body assignment in one segment to agree with assignment in another segment, but these will be synchronized across segments in a later stage. The anchor frames are, by design, good frames for later merging of traces from consecutive segments.

### Computing a maximum probability trace

#### A hidden Markov model for multi-animal tracking

A Markov model is a statistical model that consists of a set of states and two key types of probabilities: transition (probability of moving from one state at time i to another state at time i+1) and emission (probability that state j will emit some observable product at time i). *Hidden* Markov models (HMMs) are used to label observed sequential data that are presumed to have been emitted by the Markov model: the true path through the set of states is treated as unknown (hidden), and the task is to infer the series of states that is responsible for the observed data by computing a path through the states that has maximum probability under the transition and emission probabilities.

DIPLOMAT treats each (x,y) coordinate at time i as a state S(i,x,y). The probability that a state “emits” a specific body part b is taken from pseudo-probabilities produced by the pose estimation neural network: P(i,b,x,y) is the probability that body part b is observed at coordinate (x,y) and time i. State S(i,x,y) has non-zero transition probabilities to all states associated with time $i+1\;:\;(S\left(i+1,x^{\prime }y^{\prime }\right)\;\text{for}\;\text{all}\;x^{\prime },y^{\prime })$, and no others; these transition probabilities follow a distance-dependent decaying function. DIPLOMAT can produce a maximum-probability trace for an individual body part through this model by following the Viterbi algorithm, in which all possible traces are considered and stored through a standard recurrence (given below), and a maximum probability path is recovered from the stored Viterbi matrix.

The between-frame movement transition probabilities are defined as a modified Gaussian distribution. The basic form of the distribution has a mean of zero and a standard deviation that is automatically determined as follows: for each instance of body part a in frame i, identify the nearest instance of a in frame i+1 and consider that to be the matched entry; consider this to be the distance moved for that instance from frame i. The mean and standard deviation are computed after accumulating values over all instances of a and all frames in the video. This distribution is designed to allow movement between frames such that small movements are more likely than large movements, and very large movements are effectively impossible. A simple Gaussian will inappropriately penalize small movement relative to non-movement, so DIPLOMAT establishes a small radius of movement that should be treated as equally probable, computes the sum of the Gaussian probabilities of all pixel steps within that radius, then evenly distributes the sum across those pixel steps. The resulting transition probability function $T\left(x^{\prime },y^{\prime },x,y\right)$ is a flat-topped Gaussian-like distribution such as the one in Figure 4A.

We note that this is an atypical emission probability model in which there exist a large number of states specific to each time point, and each state’s emission probability is connected to the emission probability of each other state at the same time point. Moreover, the emission probabilities are not baked into a predefined model, but are instead the result of a complex data-dependent function: a neural network. One notable disconnect between the model and reality is that a body part can only truly appear in one position, but the model allows for the possibility that it may appear (with some probability) in many (or zero) positions. Even so, the resulting model, with defined transition probabilities and position-specific emission probabilities, is a legal HMM, and can serve as the basis of a maximum probability trace.

#### Maximum probability trace for a single individual

For a single individual k within a single segment starting at frame s and ending at frame e, a maximum probability trace is computed by establishing each body part b at the location (x,y) in frame s with the highest probability within the cluster for b that is assigned to individual k. From this start point, a probability $V_{\left(i,b,k\right)}(x,y)$ is computed for path for body part b for individual k running through position (x,y) in frame i, based on the recurrence:

(2) $$V_{\left(i,b,k\right)}(x,y)=P_{\left(i,b\right)}(x,y)\;*\;{max}_{\left(x^{\prime },y^{\prime }\right)}\left(V_{\left(i-1,b,k\right)}\left(x^{\prime },y^{\prime }\right)\;*\;T\left(x^{\prime },y^{\prime },x,y\right)(x,y)\right)$$

where $P_{\left(i,b\right)}(x,y)$ is the pseudo-probability (from DLC/SLEAP) of body part b at pos (x,y) in frame i, and $T\left(x^{\prime },y^{\prime },x,y\right)$ is the probability of a body part moving from ($x^{\prime },y^{\prime }$) to (x,y) between two consecutive frames. This recurrence is completed up to frame e, with a the maximum probability path recovered using the Viterbi algorithm.

By default, this simple recurrence is modified by incorporating an additional skeletal modifier that seeks to keep together all body parts for a single individual through the course of a trace. When computing the trace of a single instance of body part b in individual k, the influence in frame i from one other body part a is determined by considering all possible positions (w,z) of a for k in frame i-1, and computing the skeletal value from equation 1. Note that the set of $K\left(a_{wz},b_{xy}\right)$ values is not a proper probability distribution (the values do not sum to 1.0), but can be treated as such for the purpose of computing a maximum probability trace, since values could be linearly scaled to produce normalized probabilities.

The full trace recurrence computes an α-weighted mixture of body movement (equation 2) and geometric mean over skeletal influences among the set $A_{b}$ of body parts connected to b, where the default α=0.05 moderates the skeletal influence (note: the set of skeletal connections can be configured, or can default to a completely interconnected graph):

(3) $$V_{\left(i,b,k\right)}^{\prime }(x,y)=P_{\left(i,b\right)}(x,y)\cdot \left\{\begin{array}{l} {max}_{\left(x^{\prime },y^{\prime }\right)}\left(V_{\left(i-1,b,k\right)}^{\prime }\left(x^{\prime },y^{\prime }\right)\cdot T\left(x^{\prime },y^{\prime },x,y\right)\right).\\ {\left(\prod_{a\in A_{b}}{max}_{\left(w,z\right)}\left(V_{\left(i-1,a,k\right)}^{\prime }(w,z)\cdot K\left(a_{wz},b_{xy}\right)\right)\right)}^{\alpha /\left|A_{b}\right|} \end{array}\right.$$

### Mutually-independent maximum probability traces

Tracking multiple individuals in a segment of the recording can be achieved by repeating the process from the previous section for each individual: for individual k within the segment, begin with the body part positions assigned to k in the anchor frame, and trace a maximum probability skeleton-adjusted path for those body parts to the end of the segment. Due to transient physical proximity of individuals and subtle flaws in the movement model, independent calculation of paths can lead to multiple paths converging, so that multiple individuals overlap on the same positions in the video. In order to force individuals to remain physically separated, DIPLOMAT modifies the Viterbi calculation to enforce mutually independent traces. For each body part type b and for each pixel (x,y) in frame i, DIPLOMAT identifies the instance k with maximum $V_{\left(i,b,k\right)}(x,y)$, and considers this to be the dominant individual for that pixel. This is appropriate because individual k will always dominate others individuals for any path leading through (x,y) at frame i. For all other individuals $h,V_{\left(i,b,h\right)}(x,y)$ is set to zero, to ensure that paths for those individuals do not collapse into a pixel where they are dominated by k.

### Occluded states

In some frames, a body part may be entirely missed by the pose prediction model. Because DIPLOMAT identifies a most probable path through only pixels with non-trivial probability, the result will be that the natural path is lost and the body part must jump to some other location. To avoid this tracing error, DIPLOMAT makes use of “occluded states”, which model the unobserved presence of a body part in a pixel. The probability held in the occluded state for body part b at pixel (x,y) in frame i is $O_{\left(i,b\right)}(x,y)=P_{\left(i-1,b\right)}(x,y)\cdot t_{o}$, where $t_{o}$ is the probability of transitioning to an occluded state. Return from occluded state to visible state is possible, and adds an additional competitor $O_{\left(j-1,b\right)}(x,y)$ to the *max* function of $V_{\left(j,b\right)}(x,y)$.

### Stitching trace segments

Within each segment, the result of the above tracking steps is a collection of n sets of body part traces. Importantly, the identity associated with each set is arbitrary, since assignments were made on anchor frames with no communication between segments. Time-adjacent segments are connected by an anchor frame, which by design is fairly-well separable – this allows straightforward strategy for synchronizing identities across segments together. Adjacent segments are joined by considering positions of body parts in the final frame of the earlier segment and the first frame of the later segment. Typically, matching of individuals from one frame to the next is a straightforward matter of linking individuals that are closest to each other across frames, but movement can sometimes confound a greedy approach. For a more robust stitching is achieved in two steps: (1) for n individuals, an *n*-by-*n* distance matrix is computed, with the distance of individual i in the preceding frame to individual j in the following frame computed as the sum of body part Euclidean distances between the frames, and (2) the Hungarian assignment algorithm is applied to optimally match all individuals. Following pairwise stitching of individuals across segments, DIPLOMAT modifies all individual identities in the second segment to match the assignment from the segment containing the highest quality anchor frame.

### Interact

Because automated tracking assignments are likely to make mistakes across long video recordings, DIPLOMAT is released with a graphical user interface (GUI) that implements what we call the *Interact* stage of DIPLOMAT, and simplifies the task of manually correcting automated errors. An important feature of the GUI is its tight integration with the DIPLOMAT tracking algorithm: after modifying body-part locations on a few frames, a user may cause the Viterbi tracing algorithm to be re-run while accounting for the changes. Internally, DIPLOMAT modifies probability matrix to place 100% confidence in the manual label (i.e. when the user has placed body part b at position (x,y) of frame i, then P(i,b,x,y)==1 and P(i,b,w,z)==0 for all (w,z)≠(x,y)), then re-runs Viterbi. Because P(i,b,x,y)==1, the trace must go through (x,y) and un-modfied errors in neighboring frames are likely to follow the corrected assignments.

Following completion of the automated trace, the *Interact* GUI helps the user to identify tracking errors by computing two measures for each frame that may indicate an increased risk of labeling errors: (1) maximum jump distance for any body part in the video (because large changes in location for an individual may indicate a body swap) and (2) a measure of the variance (entropy) of distances moved by various body parts in the video (hi entropy of movement distances suggests that one or more parts may have swapped to a new body). The user may step forward through peak values of these indicators in order to quickly find errors requiring manual intervention. Once a misplaced body part has been identified, DIPLOMAT optionally allows a mode that snaps a single body part or entire set of parts to probability peaks near the mouse-controlled position, and also allows a simple key interface to enumerate frames while controlling part position. These functions, combined with Viterbi retracing, enable faster adjustment tracking errors.

### Validation methods

Fifteen, 1 min mouse triplet MABe “training” videos were used for ground truth labeled frames to train both SLEAP and DLC models (27,000 frames). All models were trained on the same data, with the 5 body part model (BPM-5) using only a subset of the body parts available in the MABe benchmark. Training and testing were performed using a system with two Xeon E5–2695v4 14-core processors, 192GB system RAM, and two Nvidia P100 GPUs with 16GB RAM. Since DIPLOMAT was designed to run on the same models as are used for DLC and SLEAP, no further optimizations were performed and all measures were only considered relative–i.e., DLC was only compared with DIPLOMAT-DLC, and SLEAP only with DIPLOMAT-SLEAP (comparisons between DLC and SLEAP were considered invalid). Lack of precise optimization was also advantageous for ensuring that a sufficiently large subset of videos included at least some body swaps, to avoid floor effects and ensure meaningful comparisons.

## Figures and Tables

**Table 1.** Means and Wilcoxon signed-rank test p-values across all **individual body parts**. DIPL_DLC_ represents results using DIPLOMAT on top of DLC per-frame inferences, while DIPL_SLEAP_ uses SLEAP inferences. Red text indicates significant (P≤0.05) improvement with DIPLOMAT.

	DLC	DIPL_DLC_	rank test p-value	SLEAP	DIPL_SLEAP_	rank test p-value

**10 body part:**						
body-swaps	60.3	7.3	3.2 x 10^−4^	23.9	5.2	2.0 x 10^−3^
ID recall	0.79	0.91	5.7 x 10^−7^	0.23	0.76	1.1 x 10^−10^
ID precision	0.93	0.91	0.062	0.28	0.82	1.1 x 10^−10^
MOTP	17.1	18.4	0.017	39.5	15.0	2.4 x 10^−4^

**5 body part:**						
body swaps	28.7	6.2	0.036	14.4	3.5	0.14
ID recall	0.77	0.87	6.7 x 10^−7^	0.19	0.74	1.1 x 10^−10^
ID precision	0.92	0.88	0.0047	0.21	0.78	1.1 x 10^−10^
MOTP	17.4	19.9	5.6 x 10^−5^	30.7	15.2	0.0011

**Table 2.** Means and Wilcoxon signed-rank test p-values for **whole body** (i.e., mean of all body parts). Headers as in the previous table. Red text indicates significant (P≤0.05) improvement with DIPLOMAT.

	DLC	DIPL_DLC_	rank test p-value	SLEAP	DIPL_SLEAP_	rank test p-value

**10 body part:**						
body swaps	1.8	0.29	0.044	3.8	0.4	0.0035
ID recall	0.86	0.98	5.8 x 10^−6^	0.89	0.93	0.0085
ID precision	0.87	0.98	5.8 x 10^−6^	0.89	0.93	0.018
MOTP	15.4	17.2	0.46	58.6	40.5	1.8 x 10^−4^

**5 body part:**						
body swaps	1.6	0.38	0.052	4.7	0.56	0.0015
ID recall	.84	.96	4.5 x 10^−7^	0.71	0.83	5.5 x 10^−4^
ID precision	0.84	0.96	4.5 x 10^−7^	0.73	0.82	0.0045
MOTP	14.7	21.0	0.24	170	38.6	6.0 x 10^−10^
